# Daily Morning Blue Light Therapy for Post-mTBI Sleep Disruption: Effects on Brain Structure and Function

**DOI:** 10.3389/fneur.2021.625431

**Published:** 2021-02-05

**Authors:** Adam C. Raikes, Natalie S. Dailey, Brittany Forbeck, Anna Alkozei, William D. S. Killgore

**Affiliations:** ^1^Center for Innovation in Brain Science, University of Arizona, Tucson, AZ, United States; ^2^Social, Cognitive, and Affective Neuroscience Lab, University of Arizona, Tucson, AZ, United States

**Keywords:** concussion, phototherapy, daytime sleepiness, fatigue, gray matter volume, functional connectivity

## Abstract

**Background:** Mild traumatic brain injuries (mTBIs) are associated with novel or worsened sleep disruption. Several studies indicate that daily morning blue light therapy (BLT) is effective for reducing post-mTBI daytime sleepiness and fatigue. Studies demonstrating changes in brain structure and function following BLT are limited. The present study's purpose is to identify the effect of daily morning BLT on brain structure and functional connectivity and the association between these changes and self-reported change in post-mTBI daytime sleepiness.

**Methods:** A total of 62 individuals recovering from a mTBI were recruited from two US cities to participate in a double-blind placebo-controlled trial. Eligible individuals were randomly assigned to undergo 6 weeks of 30 min daily morning blue or placebo amber light therapy (ALT). Prior to and following treatment all individuals completed a comprehensive battery that included the Epworth Sleepiness Scale as a measure of self-reported daytime sleepiness. All individuals underwent a multimodal neuroimaging battery that included anatomical and resting-state functional magnetic resonance imaging. Atlas-based regional change in gray matter volume (GMV) and region-to-region functional connectivity from baseline to post-treatment were the primary endpoints for this study.

**Results:** After adjusting for pre-treatment GMV, individuals receiving BLT had greater GMV than those receiving amber light in 15 regions of interest, including the right thalamus and bilateral prefrontal and orbitofrontal cortices. Improved daytime sleepiness was associated with greater GMV in 74 ROIs, covering many of the same general regions. Likewise, BLT was associated with increased functional connectivity between the thalamus and both prefrontal and orbitofrontal cortices. Improved daytime sleepiness was associated with increased functional connectivity between attention and cognitive control networks as well as decreased connectivity between visual, motor, and attention networks (all FDR corrected *p* < 0.05).

**Conclusions:** Following daily morning BLT, moderate to large increases in both gray matter volume and functional connectivity were observed in areas and networks previously associated with both sleep regulation and daytime cognitive function, alertness, and attention. Additionally, these findings were associated with improvements in self-reported daytime sleepiness. Further work is needed to identify the personal characteristics that may selectively identify individuals recovering from a mTBI for whom BLT may be optimally beneficial.

## Introduction

Mild traumatic brain injury (mTBI), or concussion, occurs when an individual experiences a blow to the head or other mechanical force that results in altered cognition and/or brief loss of consciousness. The short- and long-term effects of mTBIs are of significant concern across the spectrum of sports, military, and public health. In light of the conservatively estimated 3 million mTBIs reported to emergency rooms each year (1, 2) those sustained by athletes (3) and military Service members (4) that are cared for by embedded medical teams, the need for effective treatments is great. While considerable work is being done to identify biomarkers of injury (5–7) and improve clinical diagnoses (8–10), there is comparatively little in the way of efficacious treatments (11–14).

Among the myriad of neurobiological consequences faced by individuals who have sustained a mild traumatic brain injury, sleep disruption is among the most common and the most persistent (15–19). Up to 90% of individuals who sustain a mTBI report some form of sleep disruption—including insomnia, frequent wakefulness after sleep onset, and a general sense of poor sleep quality (19–24)—or associated complaints of increased daytime fatigue or sleepiness that interferes with one or more activities of daily living (25–28). These sleep-related complaints often go untreated and may persist for months to years post-injury.

Post-mTBI sleep disruption is also associated with slowed recovery (29–32), exacerbated symptom presentation (29, 31), and degraded overall functioning (cognitive, motor, emotional) (33–37). Notably, a reciprocal cycle appears to exist where daytime fatigue and sleepiness predispose individuals to future mTBIs, while mTBIs predispose individuals to future daytime fatigue and sleepiness (27). Consequently, identifying and developing effective treatments to deal with sleep-related disruptions is necessary to support high-quality academic, job, and sport performance. While there is evidence that pharmacologic intervention may be beneficial for some individuals following a mTBI (38–40), there is some suggestion that these may not be the best first course of treatment (41) and it is likely that non-pharmacologically driven treatments offer the added benefit of lower risk for substance dependence.

Among available non-pharmacologic treatments for sleep-related disruptions, morning blue light therapy (BLT) has been shown to be effective in reducing daytime fatigue in individuals recovering from a mTBI (42–44). Additionally, this work suggests that these effects on daytime fatigue may be associated with shifts in circadian phase in some patients (43). While these outward effects on daytime functioning are encouraging, the associated effects of blue light therapy on the brain in those recovering from a mTBI has not been extensively examined.

Work from our group has focused on using multimodal neuroimaging to examine both the acute effects of blue light on cognitive function and alertness, as well as the effects of treatment in those recovering from a mTBI. Our work suggests that – in comparison to amber light exposure—a single, short duration (30 min) blue light exposure modulates anterior cingulate activation in anticipation tasks (45), dorso- and ventrolateral prefrontal cortex activation in working memory tasks (46), and may improve neural efficiency during interference tasks (47) while being associated with improved task performance on anticipation, working memory, and verbal memory tasks (45, 46, 48).

Recently, we completed two separate randomized clinical trials using morning blue light therapy (BLT) as a treatment for mTBI-related sleep disruption vs. an amber light control condition. Across both trials, BLT was associated with reduced self-reported daytime sleepiness at the end of six of weeks of treatment (43, 44); a circadian phase advance and increased midday sleep onset latency (43); and improved actigraphically-measured nighttime sleep quality (44). Neuroimaging evidence from the first trial indicated that BLT was associated with increases in bilateral thalamic volume, particularly in the pulvinar region (43); improved functional and structural connectivity between the thalamus and areas of the parietal cortex (43); and altered white matter diffusion characteristics in white matter tracts passing the through the thalamus, corpus callosum, and left anterior corona radiata (43, 49). These neuroimaging findings suggest that altered brain structure and function in regions and circuits subserving alertness, attention, and cognitive control may be facilitated by improved nighttime sleep and reduced daytime sleepiness.

What remains unknown are the broader effects of BLT on brain structure and function following a mTBI. The effects on thalamic volume and connectivity described above were observed in a small sample (total *n* = 31) using targeted follow-up analyses (e.g., thalamic volume changes were observed using voxel-based morphometry and subsequently used as a seed region for examining function and structural connectivity changes) (43). The purpose of the present study is to expand these neuroimaging findings by (A) combining the samples from both trials and (B) applying an atlas-based approach to both gray matter volumetry and functional connectivity to explore the effects of BLT, as opposed to amber light therapy (ALT), in individuals recovering from a mTBI. We hypothesized that, consistent with prior findings, BLT would be associated with increased volume in and connectivity between cortical and subcortical regions involved in task-related attention and cognitive control.

## Materials and Methods

### Participants

Sixty-two individuals were recruited across two conceptually linked studies on the effects of BLT on sleep-related outcomes following mTBI. These studies took place in Boston, MA (study 1; *n* = 31) and Tucson, AZ (study 2; *n* = 31). Data on these two samples have been reported previously for individual samples (43, 44, 49), but the findings reported here are novel and have not been previously presented. Inclusion and exclusion criteria were the same across both studies and have been described elsewhere (43, 44, 50). Briefly, all individuals sustained a mTBI within 18 months of enrollment according to the mTBI definition consistent with the Veteran's Administration/Department of Defense guidelines (51). Individuals provided documentation of their head injury either from a medical provider, qualified third-party witness, or first responder. Qualified witness reports—including those from a coach, allied health professional, or emergency personnel—were accepted, as many mTBIs are not evaluated in a physician's office or emergency department. mTBI reports were further corroborated by the participants via the Ohio State University Traumatic Brain Injury ID self-report form (52, 53). Individuals were excluded on the basis of pre-existing medical or neuropsychiatric disorders, a history of a moderate to severe TBI, alcohol or illicit substance abuse, or contra-indications for neuroimaging. None of the participants were undergoing treatment or taking medications for sleep disorders. Complete exclusionary criteria have been previously described (43, 44). Prior to study initiation, all procedures were reviewed and approved by the Institutional Review Boards for Partner's Healthcare (study 1), the University of Arizona (study 2), and the Human Research Protections Office of the U.S. Army (both studies). All participants were fully informed of all study procedures and provided written informed consent.

### Study Procedures

Participants completed three in-lab visits separated by 1- and 6-weeks, respectively. Individuals were first screened to confirm meeting eligibility criteria and given a wrist-worn accelerometer used to quantify 24-h sleep patterns for the duration of the study [Philips Respironics Actiwatch Spectrum; data previously reported and not included here (43, 44, 50)]. Participants returned to the lab 1 week later and completed a comprehensive neuropsychological and self-report assessment battery, as well as a multimodal imaging protocol (described below). Following these procedures, individuals were randomized to either the BLT (λ ~ 469 nm; total illuminance 214 lux; total irradiance: 248 μW/cm^2^ at 50 cm; Philips goLITE BLUE, Philips Electronics, Stamford Connecticut) or ALT (λ ~ 578 nm; total illuminance:188 lux; total irradiance: 35 μW/cm^2^ at 50 cm; Philips Electronics custom light box) treatment groups. Participants were provided a corresponding light box to be used at home, based on treatment group. Treatment consisted of 6 weeks of at-home daily light box use (30 min per day within 2 h of waking). The light device was placed at arm's length at a slight angle from the participant's direction of gaze, but facing the participant enough to bathe the face and eyes with the light. Participants returned to the lab after 6 weeks of light therapy and completed identical testing and neuroimaging procedures.

### Image Acquisition

The multimodal neuroimaging protocol included the collection of structural, functional, and diffusion weighted imaging. Neuroimaging was conducted pre- and post-treatment, resulting in two sets of images for each participant. The results presented here include only the structural and functional imaging findings. With a few minor exceptions, imaging protocols were nearly identical between the two studies.

#### Study 1: Boston, MA

All imaging data were collected on a 3.0T Siemens Tim Trio (Erlanger, Germany) magnetic resonance imaging (MRI) scanner located at McLean Hospital, Belmont, MA. Imaging included a T1-weighted 3D magnetization-prepared rapid acquisition gradient echo sequence (MPRAGE; TE: 2.3 ms; TR: 2.1 s; flip angle: 12°; acquisition matrix: 256 × 256; slice thickness: 1 mm, voxel size: 1 mm^3^) and a resting-state functional MRI sequence (rsFMRI; TE: 30 ms; TR: 2 s; flip angle: 90°; acquisition matrix: 64 × 64; slice thickness: 3.5 mm; voxel size: 3.5 mm^3^). For distortion correction, a gradient echo field map sequence was also collected (TE: 4.92/7.38 ms; TR: 625 ms; flip angle: 90°; acquisition matrix: 64 × 64; slice thickness: 3.5 mm; voxel size: 3.5 mm^3^) resulting in two magnitude images and a phase difference map. The set of baseline scans were collected during the in-lab visit, prior to light therapy. The set of post-treatment scans were collected 6 weeks later during the final in-lab visit, following the completion of either BLT or ALT.

#### Study 2: Tucson, AZ

Imaging data were collected on a 3.0 Siemens Skyra (Erlanger, Germany) MRI scanner located at the University of Arizona. Imaging included a T1-weighted MPRAGE sequence (TE: 2.3 ms; TR: 2.1 s; flip angle: 12°; acquisition matrix: 256 × 256; slice thickness: 1 mm, voxel size: 1 mm) and a rsFMRI sequence (TE: 25 ms; TR: 2 s; flip angle: 90°; acquisition matrix: 84 × 84; voxel size: 2 mm^3^). For distortion correction, a gradient echo field map sequence was also collected (TE: 4.92/7.38 ms; TR: 625 ms; flip angle: 90°; acquisition matrix: 64 × 64; slice thickness: 3.5 mm; voxel size: 3.5 mm^3^) resulting in two magnitude images and a phase difference map. Baseline scans were collected prior to light therapy and post-treatment scans were collected 6 weeks later, following the completion of at-home light therapy.

### Imaging Processing

Imaging from both studies underwent the same pre- and post-processing protocol. Prior to pre-processing all data were converted from DICOM to NIFTI format using HeuDiConv (v. 0.6.0) into a Brain Imaging Dataset (BIDS) compliant format. Image quality was initially assessed using MRIQC (v 0.15.1). All participants in the present analyses had complete structural imaging datasets (one usable T1-weighted image).

#### Structural Image Processing

All T1-weighted images were processed using the Computation Anatomy Toolbox 12 (CAT12 r1450; http://www.neuro.uni-jena.de/cat/) implemented through Statistical Parametric Mapping 12 (SPM12 r7219; https://www.fil.ion.ucl.ac.uk/spm/) using MATLAB R2016A (The MathWorks Inc, Natick, MA). Prior to processing, all images were realigned to the anterior-posterior commissure axis using Convert3D (http://www.itksnap.org/pmwiki/pmwiki.php?n=Convert3D.Documentation). These realigned data were subsequently segmented using CAT12's longitudinal pipeline, which included denoising, skull stripping, three tissue type segmentation, and normalizing to Montreal Neurological Institute (MNI) space with an output resolution of 1 mm^3^. Total intracranial volume (ICV) was additionally computed using CAT12. Following normalization, gray matter volume estimates were extracted for each of 400 cortical (54), 36 subcortical (55), and 37 cerebellar (56) regions of interest (ROIs) using a concatenation of three well-validated atlases that has been previously used for similar purposes (57). Each of the ROIs was assigned to one of nine networks based on the Yeo 17-network parcellation [using overarching network names when multiple sub-networks exist; e.g., the default mode A, B, and C networks were labels as DMN; (58)] for the cortical ROIs, one subcortical network, and a cerebellar network.

#### rsFMRI Image Pre-processing

Results included in this manuscript come from preprocessing performed using fMRIPrep 20.1.3 [RRID:SCR_016216, (59, 60)], which is based on Nipype 1.5.1 [RRID:SCR_002502, (61, 62)]. The descriptions of the pre-processing steps in fMRIPrep are provided by the creators of the software under a CC0 license and reproduced here without changes (aside from formatting for references).

##### Anatomical data preprocessing

A total of 2 T1-weighted (T1w) images were found within the input BIDS dataset. All of them were corrected for intensity non-uniformity (INU) with *N4BiasFieldCorrection* (63), distributed with ANTs 2.2.0 [RRID:SCR_004757, (64)]. The T1w-reference was then skull-stripped with a Nipype implementation of the *antsBrainExtraction.sh* workflow (from ANTs), using OASIS30ANTs as the target template. Brain tissue segmentation of cerebrospinal fluid (CSF), white-matter (WM) and gray-matter (GM) was performed on the brain-extracted T1w using *fast* [FSL 5.0.9, RRID:SCR_002823, (65)]. A T1w-reference map was computed after registration of 3 T1w images (after INU-correction) using *mri_robust_template* [FreeSurfer 6.0.1, (66)]. Brain surfaces were reconstructed using *recon-all* [FreeSurfer 6.0.1, RRID:SCR_001847, (67)], and the brain mask estimated previously was refined with a custom variation of the method to reconcile ANTs-derived and FreeSurfer-derived segmentations of the cortical gray-matter of Mindboggle (RRID:SCR_002438) (68). Volume-based spatial normalization to two standard spaces (MNI152NLin2009cAsym, MNI152NLin6Asym) was performed through non-linear registration with *antsRegistration* (ANTs 2.2.0), using brain-extracted versions of both T1w reference and the T1w template. The following templates were selected for spatial normalization: *ICBM 152 Non-linear Asymmetrical template version 2009c* [RRID:SCR_008796; TemplateFlow ID: MNI152NLin2009cAsym, (69)], *FSL's MNI ICBM 152 non-linear 6th Generation Asymmetric Average Brain Stereotaxic Registration Model* [RRID:SCR_002823; TemplateFlow ID: MNI152NLin6Asym, (70)].

##### Functional data processing

For each of the two BOLD rsFMRI runs found per subject (across all sessions), the following preprocessing was performed. First, a reference volume and its skull-stripped version were generated using a custom methodology of fMRIPrep. Head-motion parameters with respect to the BOLD reference (transformation matrices, and six corresponding rotation and translation parameters) are estimated before any spatiotemporal filtering using *mcflirt* [FSL 5.0.9, (71)]. BOLD runs were slice-time corrected using *3dTshift* from AFNI 20160207 [RRID:SCR_005927, (72)]. A B0-non-uniformity map (or fieldmap) was estimated based on a phase-difference map calculated with a dual-echo GRE (gradient-recall echo) sequence, processed with a custom workflow of SDCFlows inspired by the *epidewarp.fsl* script and further improvements in HCP Pipelines (73). The fieldmap was then co-registered to the target EPI (echo-planar imaging) reference run and converted to a displacements field map (amenable to registration tools such as ANTs) with FSL's *fugue* and other SDCflows tools. Based on the estimated susceptibility distortion, a corrected EPI (echo-planar imaging) reference was calculated for a more accurate co-registration with the anatomical reference. The BOLD reference was then co-registered to the T1w reference using *bbregister* (FreeSurfer) which implements boundary-based registration (74). Co-registration was configured with six degrees of freedom. The BOLD time-series (including slice-timing correction when applied) were resampled onto their original, native space by applying a single, composite transform to correct for head-motion and susceptibility distortions. These resampled BOLD time-series will be referred to as preprocessed BOLD in original space, or just preprocessed BOLD.

The BOLD time-series were resampled into several standard spaces, correspondingly generating the following spatially-normalized, preprocessed BOLD runs: MNI152NLin2009cAsym, MNI152NLin6Asym. First, a reference volume and its skull-stripped version were generated using a custom methodology of fMRIPrep. Several confounding time-series were calculated based on the preprocessed BOLD: framewise displacement (FD), DVARS and three region-wise global signals. FD was computed using two formulations following Power [absolute sum of relative motions, (75)], and Jenkinson [relative root mean square displacement between affines, (71)]. FD and DVARS are calculated for each functional run, both using their implementations in Nipype [following the definitions by Power et al. (75)]. The three global signals are extracted within the CSF, the WM, and the whole-brain masks.

Additionally, a set of physiological regressors were extracted to allow for component-based noise correction [CompCor, (76)]. Principal components are estimated after high-pass filtering the preprocessed BOLD time-series (using a discrete cosine filter with 128 s cut-off) for the two CompCor variants: temporal (tCompCor) and anatomical (aCompCor). tCompCor components are then calculated from the top 5% variable voxels within a mask covering the subcortical regions. This subcortical mask is obtained by heavily eroding the brain mask, which ensures it does not include cortical GM regions. For aCompCor, components are calculated within the intersection of the aforementioned mask and the union of CSF and WM masks calculated in T1w space, after their projection to the native space of each functional run (using the inverse BOLD-to-T1w transformation). Components are also calculated separately within the WM and CSF masks. For each CompCor decomposition, the k components with the largest singular values are retained, such that the retained components' time series are sufficient to explain 50 percent of variance across the nuisance mask (CSF, WM, combined, or temporal). The remaining components are dropped from consideration.

The head-motion estimates calculated in the correction step were also placed within the corresponding confounds file. The confound time series derived from head motion estimates and global signals were expanded with the inclusion of temporal derivatives and quadratic terms for each (77). Frames that exceeded a threshold of 0.5 mm FD or 1.5 standardized DVARS were annotated as motion outliers. All resamplings can be performed with a single interpolation step by composing all the pertinent transformations (i.e., head-motion transform matrices, susceptibility distortion correction when available, and co-registrations to anatomical and output spaces). Gridded (volumetric) resamplings were performed using *antsApplyTransforms* (ANTs), configured with Lanczos interpolation to minimize the smoothing effects of other kernels (78). Non-gridded (surface) resamplings were performed using *mri_vol2surf* (FreeSurfer).

Many internal operations of fMRIPrep use Nilearn 0.6.2 [RRID:SCR_001362, (79)], mostly within the functional processing workflow. For more details of the pipeline, see the section corresponding to workflows in fMRIPrep's documentation.

#### Functional Connectivity

Post-processing and functional connectivity estimation from the preprocessed BOLD time series (in MNI152NLin2009cAsym space) was accomplished for each subject and session using the eXtensible Connectivity Pipeline (XCP Engine, v 1.2.3; https://github.com/PennBBL/xcpEngine). Confound regressors were collected from fMRIPrep and included the mean global, cerebrospinal fluid, and white matter signals, framewise motion (x-, y-, and z- axis translation and rotation) as well as the derivatives and quadratic expansions of these terms [36 parameter confound regressors, (80)]. Processing steps included demeaning, detrending, and temporal filtering (0.01–0.08 Hz Butterworth filter) of the time series and regressors, as well as despiking of the BOLD time-series using *3dDespike* from AFNI [RRID:SCR_005927, (72)] followed by regression using the 36 parameters. The residual BOLD time-series following regression was the averaged in each of the 473 ROIs used in estimating the gray matter volume. However, due to varying levels of field of view coverage for the cerebellum during the resting state imaging, the 37 cerebellar ROIs were ultimately excluded. Functional connectivity was then computed as the Pearson *r* correlation between each ROI, yielding a 436 × 436 functional connectivity matrix per subject per session (400 cortical ROIs, 36 subcortical ROIs). These connectivity matrices were subsequently Fisher *r-to-z* transformed to improve the normality of the distribution of the correlation coefficients. Additional quality metrics provided by XCP Engine included mean root mean square (RMS) motion estimates and node coverage.

#### Data Harmonization

A necessary consideration in the analyses of these data is the collection of imaging on different systems in different locations over the course of several years. To ensure that the present findings were not confounded by site effects, the GMV and functional connectivity data were individually harmonized using *neuroCombat* [https://github.com/Jfortin1/ComBatHarmonization, (81)] implemented in R [v. 3.6.1, (82)]. *neuroCombat* uses an Empirical Bayes approach to minimize site-level effects in neuroimaging data while preserving biological effects. This approach has successfully been used to harmonize structural, functional, and diffusion-weighted data (81, 83, 84). To ensure that effects of interest and potentially meaningful regressors were preserved, we included age, sex, number of previous mTBIs, days post-injury, and group (BLT, ALT) in the modeling. For the functional connectivity data, we additionally included mean root mean square motion from XCP Engine as a potential covariate.

### Statistical Analyses

All post-processing statistical analyses were conducted in R and Python (v. 3.7.6). All between-groups analyses were ultimately conducted using DABEST [v 0.3.0, (85)] in Python. DABEST computes effect sizes for both between-group and within-group comparisons as well as bootstrapped, bias-corrected, accelerated (BCa) 95% confidence intervals (using 20,000 bootstrap resamples) around these effect sizes. Additionally, DABEST provides traditional hypothesis testing using Welch's *t*, Student's *t*, and Mann-Whitney *U* test *p-*values as well as a permutation-based *p-*value (5,000 permutations).

#### Treatment-Related Effects on GMV

We fit an initial regression model to both baseline and post-treatment harmonized ROI GMV estimates separately to remove the effects of total intracranial volume, age, and sex and extracted the residuals to estimate covariate-adjusted ROI volumes. We then regressed baseline covariate-adjusted values against post-treatment covariate-adjusted values. The residuals from this model were then passed to DABEST to compare BLT to ALT. Results are reported as Hedges' *g* effect sizes. In reporting these findings, we employed a hierarchical approach to controlling family-wise error. First, we included only ROIs whose 95% confidence intervals did not include 0. Second, we applied false discovery rate (FDR) correction to the permutation *p*-values from DABEST and thresholded the findings at FDR corrected *p* < 0.05. Treatment-related effects on functional connectivity.

Univariate correlations were fit for each edge at baseline in the harmonized dataset in the adjusted baseline models to additionally assess age, sex, total number of prior mTBIs, days post-injury, and relative RMS motion as potential covariates. Approximately 6% of the edges were associated with either age, total number of prior mTBIs, or days post-injury and none of these correlations survived multiple comparisons correction and so no further covariate adjustment was considered. Post-treatment functional connectivity data were regressed on the baseline data to remove baseline effects. Residuals from these baseline-adjusted models were passed to DABEST to compare BLT to ALT on an edge-by-edge basis. Similar to the GMV models we report Hedges *g* effect sizes for each edge thresholded by (1) 95% confidence intervals not including 0 and (2) FDR corrected permutation *p* < 0.05. Results were plotted using in Python using the *plot_connectome* function from Nilearn [v 0.6.2; RRID:SCR_001362; (79)].

#### Exploratory Analyses

In order to further explore the neuroimaging findings in relationship to previously identified behavior outcomes, we conducted several exploratory analyses. These analyses included correlations between baseline and baseline-adjusted daytime sleepiness (ESS scores; the primary behavioral outcome previously reported) and both GMV and functional connectivity. For baseline-adjusted ESS scores, these were computed as the residual ESS scores after regressing post-treatment values on baseline values. Consistent with our prior findings, these baseline-adjusted values were computed without adjusting for additional covariates (e.g., sex, number of mTBIs, days post-injury) as none improved the models in stepwise model selection. These correlations were fit in Python using the *bootstrap-stat* (https://github.com/rwilson4/bootstrap-stat) package to compute 95% BCa confidence intervals. Findings were initially thresholded to include only correlations with 95% BCa confidence intervals not including 0. Results were visualized using BrainNetViewer [v. 1.7; RRID:SCR_009446; (86)] for GMV correlations and *plot_connectome* for functional connectivity.

These exploratory analyses were executed to provide comprehensive estimates of effects for planning future similar studies.

## Results

A total of 62 individuals (25 males) completed all study procedures across both cohorts. In general, participants were young adults (mean age: 24.7 ± 7.8 years) who were in the chronic phase of mTBI recovery (mean weeks from index injury: 36.4 ± 20.9). Across the combined sample, ESS scores approached, on average, the threshold for excessive daytime sleepiness (mean ESS: 9.2 ± 3.4; threshold for EDS = 10) (87). We previously reported baseline balance between blue and amber groups in each of the cohorts separately (43, 44).

### Treatment Effects on GMV

**Figure 1:**
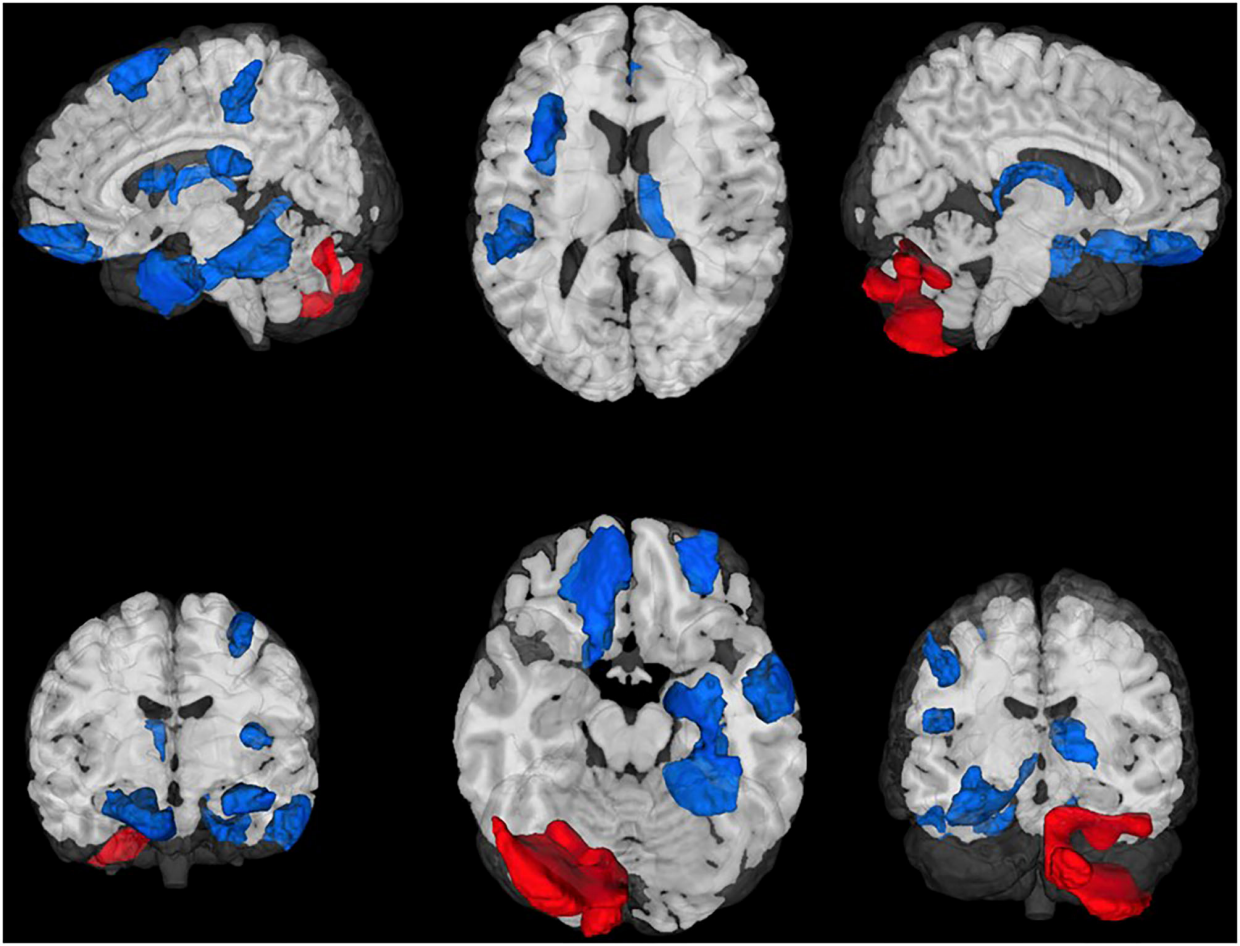
Regions of interest (ROIs) exhibiting moderate-to-large baseline adjusted differences in gray matter volume (GMV). Greater GMV was observed following blue light treatment (ROIs in blue) in 15 cortical and subcortical ROIs. Greater GMV was observed following amber light (red ROIs) in two cerebellar regions. All ROIs were false discovery rate corrected *p* < 0.05. Top Left: Lateral left view, Top Middle: Superior view; Top Right, Lateral right view. Bottom Left, anterior view; Bottom Middle: Inferior view; Bottom Right: Posterior view.

After controlling for total intracranial volume, age, sex, and baseline GMV, a total of 17 ROIs survived multiple comparisons correction (all FDR corrected *p* < 0.05) and demonstrated moderate to large differences, including greater post-treatment GMV after BLT (*n* = 15) and ALT (*n* = 2; Figure 1, Table 1). Compared to ALT, greater GMV following BLT was observed primarily in the left hemisphere in regions associated with the attention, default mode, and limbic networks. Consistent with prior findings in the Boston subset of this sample, greater GMV was also observed in the right caudal temporal and right occipital thalamic ROIs. Greater GMV following ALT compared to BLT was observed in two areas of the cerebellum.

**Table 1:** Regions of interest demonstrating baseline-adjusted differences in gray matter volume.

**Atlas**	**Region**	**Effect size**	**Interpretation**
Buckner cerebellar atlas	Cerebellum #22	0.563	Amber > Blue
	Cerebellum #34	0.554	Amber > Blue
Brainnetome atlas	Right occipital thalamus	−0.552	Blue > Amber
	Right caudal temporal thalamus	−0.557	Blue > Amber
Schaefer 400	LH_ContB_PFCd_1	−0.538	Blue > Amber
	LH_DefaultB_Temp_2	−0.615	Blue > Amber
	LH_DefaultC_PHC_2	−0.572	Blue > Amber
	LH_DorsAttnA_TempOcc_2	−0.788	Blue > Amber
	LH_DorsAttnB_PostC_4	−0.514	Blue > Amber
	LH_Limbic_TempPole_3	−0.522	Blue > Amber
	LH_SalVentAttnA_Ins_4	−0.574	Blue > Amber
	LH_SalVentAttnB_OFC_1	−0.575	Blue > Amber
	LH_SomMotB_Aud_10	−0.554	Blue > Amber
	RH_Limbic_OFC_1	−0.644	Blue > Amber
	RH_Limbic_OFC_3	−0.541	Blue > Amber
	RH_Limbic_OFC_4	−0.552	Blue > Amber
	RH_SalVentAttnA_ParMed_6	−0.485	Blue > Amber

*Regions of interest defined in the Buckner cerebellar atlas (56), Brainnetome atlas (55), and Schaefer 400 ROI 17-Network parcellation (54). Effect sizes as Hedges g*.

### Treatment Effects on Functional Connectivity

After controlling for baseline functional connectivity, a total of 3,276 edges exhibited differences in connectivity (all FDR corrected *p* < 0.05) between the BLT and ALT groups. Greater connectivity following BLT was observed in *n* = 2,028 edges while lower connectivity was observed in *n* = 1,248. Based on the network labeling for the associated ROIs, these differences included a widespread network for connections between the default mode, somatomotor, cognitive control, subcortical gray matter, as well as dorsal and ventral attention networks.

**Figure 2:**
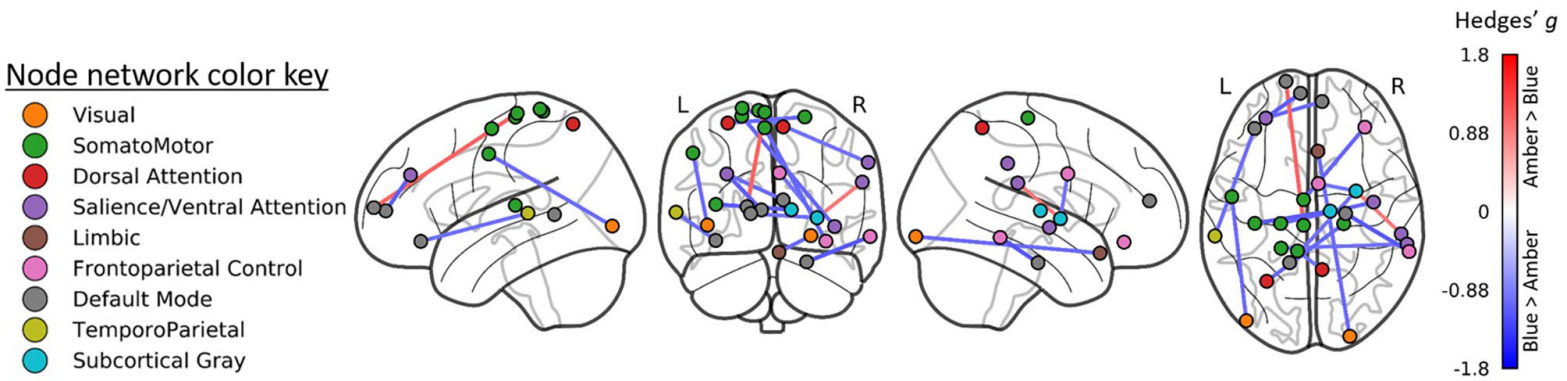
Region-to-region functional connectivity differences following treatment. Blue light treatment was associated with greater functional connectivity in 14 ROI-to-ROI connections linking the visual, default mode, somatomotor, and dorsal attention networks (all FDR *p* < 0.01).

The largest differences (FDR corrected *p* < 0.01; Figure 2) were observed in a total of 16 edges linking these same networks. These edges specifically include connections with the right thalamus, prefrontal and orbitofrontal cortices, and numerous somatomotor regions.

### Exploratory Analyses

#### Relationship Between ESS and GMV

**Figure 3:**
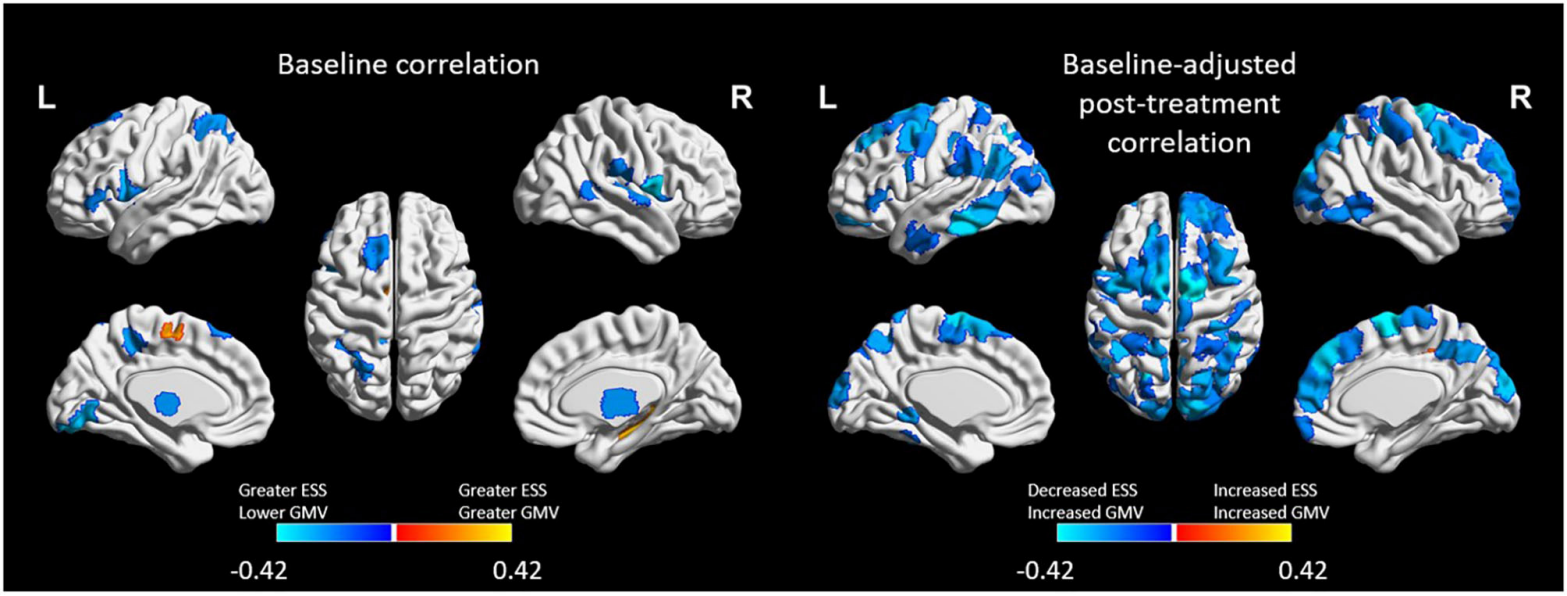
Regions of interest (ROIs) exhibiting moderate-to-large correlations (|*r*| > 0.4) between Epworth Sleepiness Scale scores and gray matter volume (GMV) at baseline (left) and at post-treatment (right). Treatment-related decreases in daytime sleepiness were associated with moderate-to-large increases in GMV in 74 ROIs. All ROIs were FDR corrected at *p* < 0.05.

At baseline, ESS scores were inversely correlated with GMV (i.e., greater ESS, lower GMV) in 20 cortical ROIs and positively associated with GMV 5 ROIs, including four subcortical ROIs. After adjusting for baseline GMV and ESS, decreased ESS scores at post-treatment were correlated with increased GMV in 15.6% of the 473 ROIs (*n* = 74; Figure 3) including the right caudal temporal thalamus. These ROIs were associated with the bilateral attention, cognitive control, visual, and default mode networks.

#### Relationship Between ESS and Functional Connectivity

Prior to treatment, ESS scores were associated with functional connectivity across a total of 5,656 edges. Those with the strongest correlations (*r* > |0.40|, *n* = 144 edges) included positive correlations (greater ESS and greater connectivity; *n* = 72 edges) within the DMN as well as between the DMN, salience and cognitive control networks (*n* = 27/72 edges) and negative correlations (greater ESS, lower connectivity; *n* = 72 edges) primarily between the DMN and subcortical gray matter as well as between the temporoparietal network and the somatomotor and ventral attention networks (*n* = 27/72 edges across these connections).

**Figure 4:**
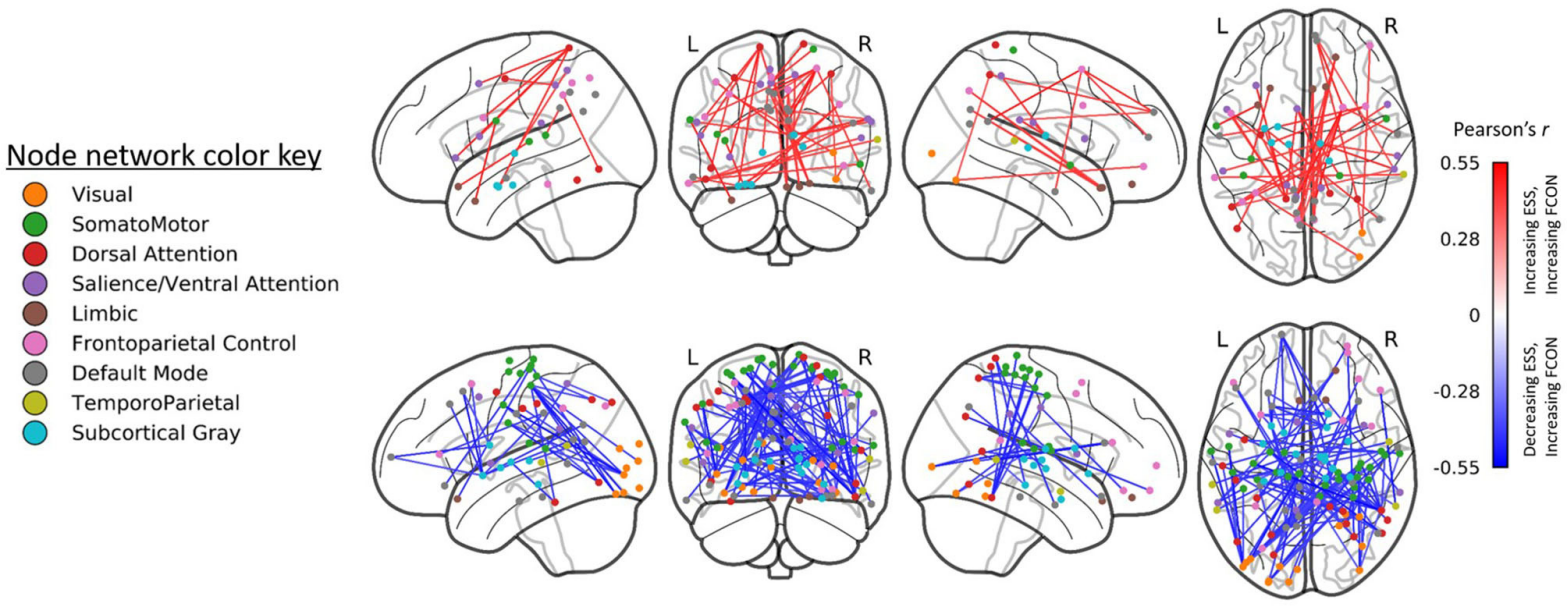
Edges (region-to-region connections) exhibiting moderate-to-large correlations (|*r*| > 0.4) between Epworth Sleepiness Scale scores and gray matter volume (GMV) at post-treatment (right). Treatment-related decreases in daytime sleepiness were associated with moderate-to-large increases in functional connectivity in 82 edges and with decreases in functional connectivity in 39 edges. All edgewise correlations were FDR corrected at *p* < 0.05.

A total 5,039 edges exhibited a change in connectivity that was associated with a change in daytime sleepiness, including 3,072 edges with increased connectivity and 1,967 with decreased connectivity. Examining those edges with the strongest correlations (*r* > |0.40|, *n* = 121 edges; Figure 4) revealed increased connectivity primarily associated with decreased ESS scores (negative correlation) in edges connecting the dorsal attention, somatomotor, and visual networks as well as the default mode, and subcortical networks (*n* = 48 edges total across these network connections; 82 total edges with negative correlations; Figure 4 bottom). Decreased connectivity was associated with decreased ESS scores in 39 edges (Figure 4 top).

## Discussion

The primary purpose of this study was to extend our prior findings on the effects of 6 weeks of 30 min daily morning BLT on brain structure and function in individuals recovering from a mTBI. Consistent with our hypotheses and prior findings, we observed increased gray matter volume and modulated functional connectivity in and between areas of the brain associated with attention, cognitive control, salience, and visual processing among those who received BLT. Increased GMV after adjusting for baseline GMV was also observed in the right thalamus, consistent with the earlier voxel-based analysis of a subset of these data (43). These changes in both GMV and functional connectivity were additionally correlated with improvement in subjective daytime sleepiness.

In particular, individuals who reported improved daytime sleepiness exhibited increased GMV in the thalamus as well as orbitofrontal and prefrontal cortices and the precuneus, among other regions. Furthermore, increased functional connectivity associated with decreases in daytime sleepiness were observed in ROI-to-ROI connections linking these regions to other areas in the attention, default mode, visual, and somatomotor networks. This is consistent with other findings suggesting that daytime sleepiness is associated with reduced thalamocortical connectivity (88). These data provide evidence that, particularly for individuals experiencing daytime sleepiness following a mTBI, blue light therapy may confer beneficial effects, in part due to changes to both structure and function in brain networks associated with alertness and attention as well as linkage between these and the default mode network.

### Blue Light Effects on GMV

The present findings extend our previous work on blue light therapy and GMV in several ways. Specifically, we provide further evidence that reduced thalamic volume is associated with daytime sleepiness following mTBI and that this volume increases following blue light therapy (as opposed to similar exposure to amber light). We additionally identified several other regions that were not previously observed in our voxel-wise analyses that may respond positively (i.e., increased GMV) to blue light therapy, including the orbitofrontal and prefrontal cortices. For both mTBI recovery and daytime sleepiness, these regions have important implications.

First, decreased thalamic volume has been observed following mTBIs, specifically in individuals with post-mTBI fatigue (89), while increased thalamic volume is positively associated with more rapid recovery from post-mTBI cognitive impairment (90). Second, both decreased thalamic volume and decreased volume in orbitofrontal and prefrontal regions has been observed in individuals with chronic insomnia, sleep loss, daytime fatigue, and daytime sleepiness (91–93). Relatedly, previous studies show that sleep loss is associated with decreases in glucose metabolism in pre-/orbitofrontal regions (94) as well as changes in the upregulation of A1 adenosine receptors which may underpin homeostatic sleep regulation (95). Given the metabolic crisis that accompanies an mTBI (96), it is possible that reduced GMV in these regions may be associated with specifically compromised glucose metabolism and sleep regulation, while increases in volume may either facilitate or be facilitated by improved sleep. Collectively, findings from those studies, as well as our present findings of decreased volume and increased ESS scores at baseline, suggest that post-mTBI sleep disruption and thalamic, orbitofrontal, and prefrontal volume may be closely linked. However, prospective studies are lacking to identify the directionality of this relationship as well as the effect of mTBI on factors associated with sleep regulation (e.g., glucose metabolism) in these areas.

### Blue Light Effects on Functional Connectivity

The present work also extends prior findings on the effects of BLT and functional connectivity. Acute exposure to blue light (even as short as 1 min) modulates task-dependent fMRI activation in regions critical to cognitive control, attention, and memory including the anterior cingulate (45) and dorso- and ventrolateral prefrontal cortex (46, 97, 98) as well as the thalamus and anterior insula (97). These effects have also been observed in completely blind individuals with brief (<1 min) exposures to blue light, indicating that these effects are not dependent on visual perception of blue light (99). Task-dependent modulation is also evident for acute blue light exposure during emotion regulation tasks (100). Additionally, and highly relevant to the present work, these acute, task-dependent modulations may be strongly influenced by both circadian phase and sleep pressure (97). Collectively, these prior findings indicate that acute blue light exposure enhances functional activation during cognitive tasks and repeated or prolonged exposure may confer lasting effects. Future work should specifically examine the similarities and differences between acute and repeated blue light exposure on both resting-state and task-dependent activation.

Further, we previously reported increased functional connectivity specifically between the left thalamus and both frontal and parietal cortical areas that was associated with decreased daytime sleepiness following treatment in this population (43). The present findings demonstrate wider influences of blue light therapy on increased coherence between visual, attention, and subcortical gray matter networks, including bilateral thalamocortical connectivity. We further demonstrate greater dissociation between internally and externally oriented networks (i.e., the dorsal vs. ventral attention networks; default mode vs. frontoparietal control networks). Critically, these effects of BLT were associated with observed decreases in daytime sleepiness.

These findings are broadly in line with prior neuroimaging findings linking mTBI and sleep disrupted states. Decreased and disrupted thalamocortical connectivity—including dorsal attention and frontoparietal control networks—has previously been reported in individuals recovering from a mTBI who report fatigue (25, 101, 102). Additional work indicates that poor quality sleep and fatigue is associated with decreased functional connectivity within the default mode network and increased limbic network functional connectivity (including thalamocortical connectivity) in pediatric mTBI (103). Those studies also indicate that connectivity in the limbic and default mode networks normalizes in parallel with the recovery of cognitive function and sleep.

### Sleep Treatments Facilitate mTBI Recovery

The present findings agree both with our prior analyses and more broadly with a recent randomized controlled trial investigating exogenous melatonin supplementation for adolescents recovering from a mTBI (38, 43). Findings from that study on melatonin also demonstrated increases in GMV following treatment that was associated with increased sleep quality (specifically, decreased wake after sleep onset as measured with actigraphy), particularly in the posterior cingulate cortex. Changes in WASO were also associated with increased functional connectivity between the default mode network and attention, visual, and somatosensory networks as well as an overall increase in whole brain functional connectivity (38).

While the exact mechanisms by which either blue light therapy or melatonin would alter brain structure and function remain to be fully elucidated, we posit that this is due primarily to effects on sleep, rather than directly the result of treatment. Recent work suggests that sleep is a critical component of synaptic plasticity (104, 105), and sleep is critical for the formation of oligodendrocyte precursor cells, which form the basis of the myelin sheath (106), which is often damaged in mTBI. Both blue light therapy (as a daytime melatonin suppressor) and melatonin supplementation (as a nighttime sleep-promotor) exert potent effects on circadian rhythm and sleep regulation (107–111). Therefore, it is likely that treatment-mediated changes in circadian rhythm and sleep enable plasticity and modulation of resting-state connectivity in the recovery from a mTBI, which may in turn facilitate clinical recovery, in keeping with the observed relationship between clinical outcomes and functional connectivity normalization. Future work is necessary to more conclusively determine the mechanisms by which these effects are observed.

### Limitations

There are several limitations that should be noted in interpreting the present findings. First, we had limited capability to fully ensure treatment compliance and dose. Treatment was completed by participants in their home and required them to position the light box within their peripheral vision each day for 6 weeks. However, across both studies self-reported compliance was high (>80% of treatment sessions completed) and the individuals with the lowest compliance were those who had missing or late recording of treatments. These lapses in self-reporting do not necessarily mean that treatment was not completed, nor does high reporting compliance necessarily indicate strict adherence or truthful reporting. The data here were analyzed with an intention-to-treat design, so that all cases with available data were included, regardless of treatment compliance. Future work in this area should endeavor to develop methods for quantitatively and objectively determining light box treatment compliance.

Second, we enrolled participants ranging from 5 to 80 weeks post-injury. Spontaneous recovery from a mTBI has been noted at least up to 12 weeks post-injury (112) and so some changes here may be attributable to post-injury timing. However, the relationship between weeks post-injury and ESS scores was neither strong nor statistically significant and so the relative impact of treatment timing may be small. Third, no clinical reads were performed and so the presence of unidentified brain pathology (e.g., vascular lesions) may have affected the present findings. However, our sample is comprised of young adults all with diagnosed injury no more severe than mild, so we consider the likelihood of this potential confound to be small.

Fourth, this was not a prospective study and we did not include a non-injured control group as a baseline or post-treatment reference. Therefore, we are unable to determine whether BLT brings individuals recovering from a mTBI closer to a pre-injury or uninjured state from a neuroimaging perspective. However, the purpose the trial was neither to establish the effects of mTBIs on brain structure or function (a baseline comparison to self or an uninjured control group) nor to demonstrate that blue light therapy normalizes to a pre-injury or non-injured state. Rather, the overall purpose of the trial was to demonstrate that, in those individuals self-reporting adverse sleep-related outcomes following injury, blue light therapy is a viable treatment alternative for reducing daytime sleepiness and improving sleep (quantity and quality) as we have previously reported (43, 44), with the present findings as secondary outcomes. Future prospective studies should examine the capacity of BLT to minimize both the neurophysiological effects and long-term sleep disruption stemming from mTBIs relative to pre-injury or uninjured states.

## Conclusion

For individuals reporting daytime sleepiness following a mild traumatic brain injury, BLT is a non-invasive treatment option that may help to facilitate neural plasticity and hasten recovery. Here, we demonstrated moderate to large increases in both gray matter volume and functional connectivity following blue light therapy in areas previously associated with both sleep regulation and daytime cognitive function, alertness, and attention. Further work is needed to more completely elucidate the exact mechanisms of these changes as well as precision medicine factors that may selectively identify individuals in whom the greatest benefits may be seen. These data add to a growing body of research suggesting that morning BLT may facilitate structural and functional brain recovery among some individuals who have sustained a mTBI.

## Data Availability Statement

The raw data supporting the conclusions of this article will be made available by the authors, without undue reservation.

## Ethics Statement

The studies involving human participants were reviewed and approved by Partners Healthcare, The University of Arizona, and The U.S. Army Human Protections Office. The patients/participants provided their written informed consent to participate in this study.

## Author Contributions

AR conducted the analyses and drafted the initial manuscript. ND revised the manuscript. BF and AA assisted with data collection, study implementation, and manuscript preparation. WDSK designed the study, analyzed the data, and assisted with manuscript preparation and revision. All authors contributed to the article and approved the submitted version.

## Conflict of Interest

The authors declare that the research was conducted in the absence of any commercial or financial relationships that could be construed as a potential conflict of interest.
